# Standardization of electroencephalography for multi-site, multi-platform and multi-investigator studies: insights from the canadian biomarker integration network in depression

**DOI:** 10.1038/s41598-017-07613-x

**Published:** 2017-08-07

**Authors:** Faranak Farzan, Sravya Atluri, Matthew Frehlich, Prabhjot Dhami, Killian Kleffner, Rae Price, Raymond W. Lam, Benicio N. Frey, Roumen Milev, Arun Ravindran, Mary Pat McAndrews, Willy Wong, Daniel Blumberger, Zafiris J. Daskalakis, Fidel Vila-Rodriguez, Esther Alonso, Colleen A. Brenner, Mario Liotti, Moyez Dharsee, Stephen R. Arnott, Kenneth R. Evans, Susan Rotzinger, Sidney H. Kennedy

**Affiliations:** 1 Centre for Addiction and Mental Health, 1001 Queen St. W, Toronto, ON M6J 1A8 Canada; 2 Department of Psychiatry, University of Toronto, 250 College Street, 8th floor, Toronto, ON M5T 1R8 Canada; 3 Institute of Medical Science, Faculty of Medicine, University of Toronto, Medical Sciences Building, 1 King’s College Circle, Toronto, ON M5S 1A8 Canada; 4 Institute of Biomaterial and Biomedical Engineering, Rosebrugh Building, Room 407, 164 College St, Toronto, ON M5S 3G9 Canada; 5 The Edward S. Rogers Sr. Department of Electrical & Computer Engineering, University of Toronto, 10 King’s College Road, Toronto, ON M5S 3G4 Canada; 6 University Health Network, 399 Bathurst Street, Toronto, ON M5T 2S8 Canada; 7 University of British Columbia and Vancouver Coastal Health Authority, 2255 Wesbrook Mall, Vancouver, BC V6T 2A1 Canada; 8 McMaster University, and St. Joseph’s Healthcare Hamilton, 1280 Main Street West, Hamilton, ON L8S 4L8 Canada; 9 Queen’s University, Providence Care, Mental Health Services, 752 King Street West, Kingston, ON K7L 4X3 Canada; 10 Loma Linda University, 24851 Circle Dr, Loma Linda, CA 92354 USA; 11 Simon Fraser University, 8888 University Dr, Burnaby, BC V5A 1S6 Canada; 12 Indoc Research, 258 Adelaide St. East, Suite 200, Toronto, ON M5A 1N1 Canada; 13 Rotman Research Institute at Baycrest Centre, 3560 Bathurst Street, Toronto, ON M6A 2E1 Canada; 14 Department of Pathology and Molecular Medicine, Queen’s University, 88 Stuart Street, Kingston, ON K7L 3N6 Canada; 15 School of Mechatronic Systems Engineering, Simon Fraser University, 250-13450 102 Avenue, Surrey, BC V3T 0A3 Canada; 16 St. Michael’s Hospital, 193 Yonge St, Toronto, ON M5B 1M4 Canada

## Abstract

Subsequent to global initiatives in mapping the human brain and investigations of neurobiological markers for brain disorders, the number of multi-site studies involving the collection and sharing of large volumes of brain data, including electroencephalography (EEG), has been increasing. Among the complexities of conducting multi-site studies and increasing the shelf life of biological data beyond the original study are *timely* standardization and documentation of relevant study parameters. We present the insights gained and guidelines established within the EEG working group of the Canadian Biomarker Integration Network in Depression (CAN-BIND). CAN-BIND is a multi-site, multi-investigator, and multi-project network supported by the Ontario Brain Institute with access to Brain-CODE, an informatics platform that hosts a multitude of biological data across a growing list of brain pathologies. We describe our approaches and insights on documenting and standardizing parameters across the study design, data collection, monitoring, analysis, integration, knowledge-translation, and data archiving phases of CAN-BIND projects. We introduce a custom-built EEG toolbox to track data preprocessing with open-access for the scientific community. We also evaluate the impact of variation in equipment setup on the accuracy of acquired data. Collectively, this work is intended to inspire establishing comprehensive and standardized guidelines for multi-site studies.

## Introduction

The common global interest in discovering early predictors and interventions for a host of debilitating brain disorders has led to an increasing number of collaborative multi-site research studies and collection of large volumes of biological data. To this end, big data informatics platforms and technological tools have emerged to empower the sharing and integration of biological data across projects, laboratories, and countries. Examples of these initiatives include: large-scale global initiatives to integrate neuroimaging and genetic data across neuropsychiatric pathologies from numerous laboratories such as the ENIGMA (Enhancing NeuroImaging Genetics through Meta-Analysis (ENIGMA) Consortium)^1^; multicentre clinical trials aimed at identifying predictors of response to treatments in specific pathologies such as EMBARC (Establishing Moderators and Biosignatures of Antidepressant Response in Clinical Care^2^); and multi-site, multi-investigator, and multi-project initiatives involving the collection of a host of biological data aimed at transforming treatment of specific pathologies such as the Canadian Biomarker Integration Network in Depression (CAN-BIND)^3, 4^ initiative of the Ontario Brain Institute^5^. Historically, genetic and structural magnetic resonance imaging (MRI) data have been the primary focus of most biological big data consortiums. However, a growing understanding of the pivotal role of change in brain function and dynamicity in various pathologies has led to the inclusion of functional neuroimaging data in many multi-site studies. Particularly, the real world application (e.g., portability and cost) of scalp-recorded electrophysiological signals through electroencephalography (EEG) has led to a number of multi-site EEG studies (e.g., ref. 2, 4). Likewise, there has been a growing interest in the meta-analysis of unstructured EEG data collected across laboratories. While the establishment of standardized analytic toolboxes for genetic and structural MRI data has, over the years, reduced the need for extensive communication between investigators in multi-site studies involving such structural data^6^, additional work is required to simplify the process of data integration for functional neuroimaging or neurophysiological data such as EEG. To date, very few initiatives, guidelines, standardized toolboxes, or general study design frameworks exist to guide collection and integration of large-scale EEG data^7, 8^.

The first step in multi-site EEG data collection and integration is the identification and assessment of sources of variability that can impact the EEG outcomes. Inter-dataset variability in EEG studies can originate from errors or differences in study designs, equipment, experimental setup, data quality control procedures, or data preprocessing and analyses. Within each of these categories, factors which affect study outcomes may be obvious and controlled for at the time of study design. However, such factors may not emerge in the early phases of study design, such as when projects are designed by multiple teams of scientists or when data integration is not planned *a priori*.

The overarching aim of this paper is to provide insights and solutions that thus far have emerged from the work of the CAN-BIND EEG group and to contribute to the ongoing effort of standardized multi-site EEG studies. We describe scenarios that initiated the development of standardized methodologies and guidelines across various phases of this multi-site EEG study, from design to data dissemination. Specifically, we draw examples from the first (out of currently nine and growing) multi-site and multi-investigator CAN-BIND project focused on the identification of EEG biomarkers of response to escitalopram and aripiprazole. The study protocol of this project has been previously published and interested readers are referred to prior publications for a detailed description of the study design and rationale^3, 4^. In current article, project-specific description is mainly provided in the context of data standardization.

## Results

**Figure 1:**
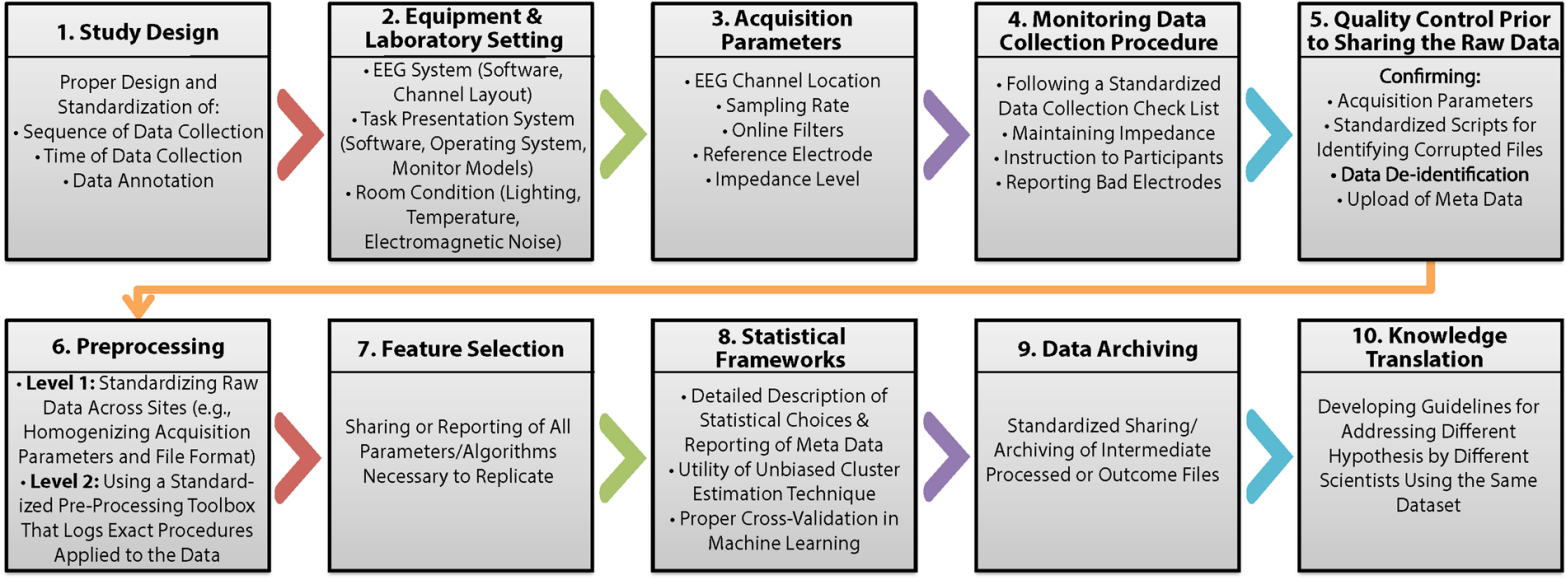
Potential Sources of Variance in Multi-Site EEG Studies. This flowchart summarizes the major phases of an EEG study in chronological order. For each phase, the diagram lists items/parameters/guidelines that need to be standardized in multi-site/multi-project/multi-investigator EEG studies.

In EEG studies, inter-dataset variability can emerge due to errors or differences in: 1) study design, 2) equipment, software and laboratory setting, 3) acquisition parameters, 4) data collection monitoring procedures, 5) quality control procedures prior to data sharing, 6) parameters and algorithms used in data pre-processing, 7) EEG feature extraction algorithms, 8) the choice of statistical frameworks, 9) data archiving processes, and 10) the method of knowledge translation (Fig. 1). Next, we will discuss the EEG-specific and general considerations listed above, as well as guidelines that we have put together to reduce bias and variability within each of these categories.

### Study design

A number of key study design considerations should be followed in multi-site EEG studies. These include: determining the proper sequence of data collection, planning for data integration, standardizing the time of day for data collection, standardization of instructions given to participants prior to EEG visits, and annotating the collected data with parameters that may differ across studies.

#### Sequence of data collection

Study protocols should describe plans for controlling the bias introduced by the sequential application of a study procedure, especially when similar study procedures are conducted using different modalities of neuroimaging procedures. For example, if participants are required to perform the same task using EEG and functional MRI (fMRI), the order of modalities may need to be randomized to not introduce systematic biases due to practice effects. Specifically, every effort should be made to keep the randomization procedure similar across sites and between patients and controls. As described next, other study designs may be considered if integrative analyses are desired.

#### Planning for data integration

Integrative analyses (in which input variables span different platforms) should be discussed and planned prior to study launch. When integrative analyses across multiple platforms (e.g., EEG and fMRI) are expected, study protocols should include methodological and statistical plans for data integration. As an example, if the aim of administering a behavioral task or condition (e.g., resting-state) using several modalities is to combine the results from all modalities into a single comprehensive outcome — for example, quantify the same neural mechanism using both EEG and fMRI — then caution should be exercised to keep the parameters of the task and experimental conditions as similar as possible between the modalities. This can be challenging in the case of a behavioral task, as the rate (frequency of stimuli presentation) and number of stimuli presented are tailored for each modality specifically (e.g., more stimuli are presented for EEG to increase the signal to noise ratio with lower rates of presentation used in fMRI). Moreover, the resting-state condition in MRI studies involves participants lying down on the MRI table while subjected to loud auditory background noise of the MRI machine. In EEG, on the other hand, the resting-state condition often involves an upright sitting position with no systematic exposure to loud auditory noise. These differences may lead to activation of different neural mechanisms across modalities. To minimize the possibility of activating different mechanisms, the tasks and conditions should be kept as similar as possible between the two modalities. Alternatively, investigators may consider concurrent multi-modal data collection (e.g., concurrent EEG and fMRI). However, this would require access to fMRI compatible EEG units and extensive denoising procedures to remove MRI artifact from EEG. Collectively, these issues must be discussed and planned among investigators of platforms early on and preferably prior to study launch.

#### Time of data collection

Efforts should be made to keep the time of data collection consistent across sites and within subjects. Fluctuations in circadian rhythms could impact functional data (e.g., EEG)^8^. This can lead to inter-site biases if there are differences in the time of data collection between sites. The time of EEG collection should also not significantly differ across the studied groups (e.g., patients and controls) and within groups (e.g., longitudinal assessment). This may be challenging due to variability in the availability of EEG laboratory space across the participating sites. In such instances, data must be annotated to permit post-hoc assessment for impact of “time of day” on the outcomes. Finally, as discussed next, standardized instructions should be given to participants across different sites and studies to avoid data collection under sleep deprivation. For example, a set of standardized instructions could be sent to participants prior to each study visit to remind them that they should get a full night sleep and cancel their study visit if they are unable to do so.

#### Standardization of instructions given to participants prior to EEG visits

EEG signals are composed of both trait and state components. In order to reduce the level of noise in the state component, it is crucial to develop a standard operating procedure (SOP) to instruct subjects about aspects such as sleep hygiene, caffeine intake, smoking habit, alcohol intake before EEG. Such SOP should aim at decreasing the noise caused by those parameters rather than rigid prescriptive rules. In this regard, compliance with SOP should be monitored in a way that promotes participants’ reporting of deviations from the prescription. The reporting of deviation(s) from the SOP should be carefully and systematically documented in data annotation procedures described next.

#### Data annotation

It is of the essence to establish a clear and consistent naming convention that is strictly followed across sites. This is critical as studies may include multiple tasks and conditions, several groups and time points if longitudinal data are collected. Compliance with naming convention should be monitored throughout the study, and particularly at the onset of the study. The type and volume of technical and demographic information collected at the time of the EEG experiment could be potentially essential in accounting for non-specific effects and controlling for potential confounds. This may also enhance the probability of data reusability and integration in the future. Examples include but are not limited to: time of data collection; consumption of caffeinated drinks, over-the-counter medications (e.g., antihistamines or non-steroid anti-inflammatory drugs), or tobacco smoking immediately prior to data collection; consumption of alcoholic beverages in the last 24 hours; rigorous physical exercise or consumption of a heavy meal immediately prior to the EEG session; phase of menstrual cycle; results of urine drug tests; hours of sleep the night prior to the EEG visit; drowsiness during the experiment; and other demographic information including handedness, years of education, musicianship, sportsmanship, and bilingualism. As will be discussed next, the make and model of equipment and software used in EEG studies should also be reported. The list of metadata currently captured from multi-site EEG studies hosted on the Brain-CODE informatics platform can be found on Brain-CODE website (https://www.braincode.ca/sites/default/files/about/BraincodeStandardEEGDataCollectionForm%282015%29.pdf).

### Selection of equipment, software and laboratory setting

Ideally, every effort should be made to keep the equipment and experimental conditions consistent across sites and certainly between groups (e.g. patient vs. control), as well as within subjects (i.e. in longitudinal designs were there are multiple data points captured). This may include: EEG equipment model, EEG acquisition software, EEG task software and version number, computer operating systems, room temperature, amount and nature of lightening in the EEG laboratory and level of ambient noise (e.g., sound, electromagnetic noise). In the event that these conditions cannot be kept consistent across sites, site-specific information on these conditions should be collected, archived, and shared for post-hoc evaluation of their impact on EEG outcome measures.

EEG studies measuring neural responses time-locked to the onset of a stimulus, such as event-related potentials (ERPs), should evaluate the synchrony between the stimulus presentation system (task computer) and the EEG data recording system (EEG computer). This is to account for the time delay between the true onset of a stimulus, as seen or heard by the participant, and the onset of the stimulus marked in the EEG data file. Such timing issues may arise when the task computer takes time to initialize the hardware required for stimulus presentation. This may be due to the presence of background computer processes or various inconsistencies with the stimulus presentation software. In general, users must take the necessary precautions to account for this time delay and its variation (i.e., jitters) as a function of operating systems or hardware models involved in stimulus presentation.

We conducted a series of experiments to evaluate the amount and variation of this time delay for two modes of stimuli (auditory and visual) across two different computer models/operating systems (Lenovo ThinkCentre M72e-3660 with Windows XP and Dell Optiplex 7020 with Windows 7) (Table 1). Moreover, for the visual stimulus, we set to examine whether this delay varied across monitor models. In our experiments, the stimulus presentation software was E-prime (Psychology Software Tools, Inc.) and the EEG system was Neuroscan Synamps 2/RT (Compumedics, USA). To bypass the delays associated with operating systems and accurately measure the true onset time of stimuli, a commercially-available device, StimTracker (StimTracker, Cedrus, San Pedro, CA, USA) was used.

**Table 1:** The Time Delays in Event-Related Potential Studies Associated with an Auditory or Visual Stimulus over Different Operating Systems and Hardware Setups

Stimulus Type	Operating System	Equipment Info	Measured Delay (ms) +/− Std Dev
***Auditory***	Windows XP SP3 (32-bit System) on Lenovo ThinkCentre M72e-3660	Direct Sound – Primary Sound Driver	24.6 +/− 0.59
	Windows 7 Professional SP1 (64-bit System) On Dell Optiplex 7020	Direct Sound – Primary Sound Driver	49.3 +/− 2.93
***Visual***	Windows XP SP3 (32-bit System) on Lenovo ThinkCentre M72e-3660	Lenovo ThinkVision 19-inch Wide LED-backlit LCD monitor (LT1952pwD)	Centre: 19.3 +/− 0.91 Corner: 29.4 +/− 1.2
		Dell UltraSharp 24-inch Monitor with LED Backlight (U2412Mb)	Centre: 12.4 +/− 0.045 Corner: 20.1 +/− 0.098
	Windows 7 Professional SP1 (64-bit System) on Dell Optiplex 7020	Lenovo ThinkVision 19-inch Wide LED-backlit LCD monitor (LT1952pwD)	Centre: 18.7 +/− 1.1 Corner: 30.4 +/− 1.3
		Dell UltraSharp 24-inch Monitor with LED Backlight (U2412Mb)	Centre: 12.8 +/− 0.095 Corner: 22.4 +/− 0.37

**Figure 2:**
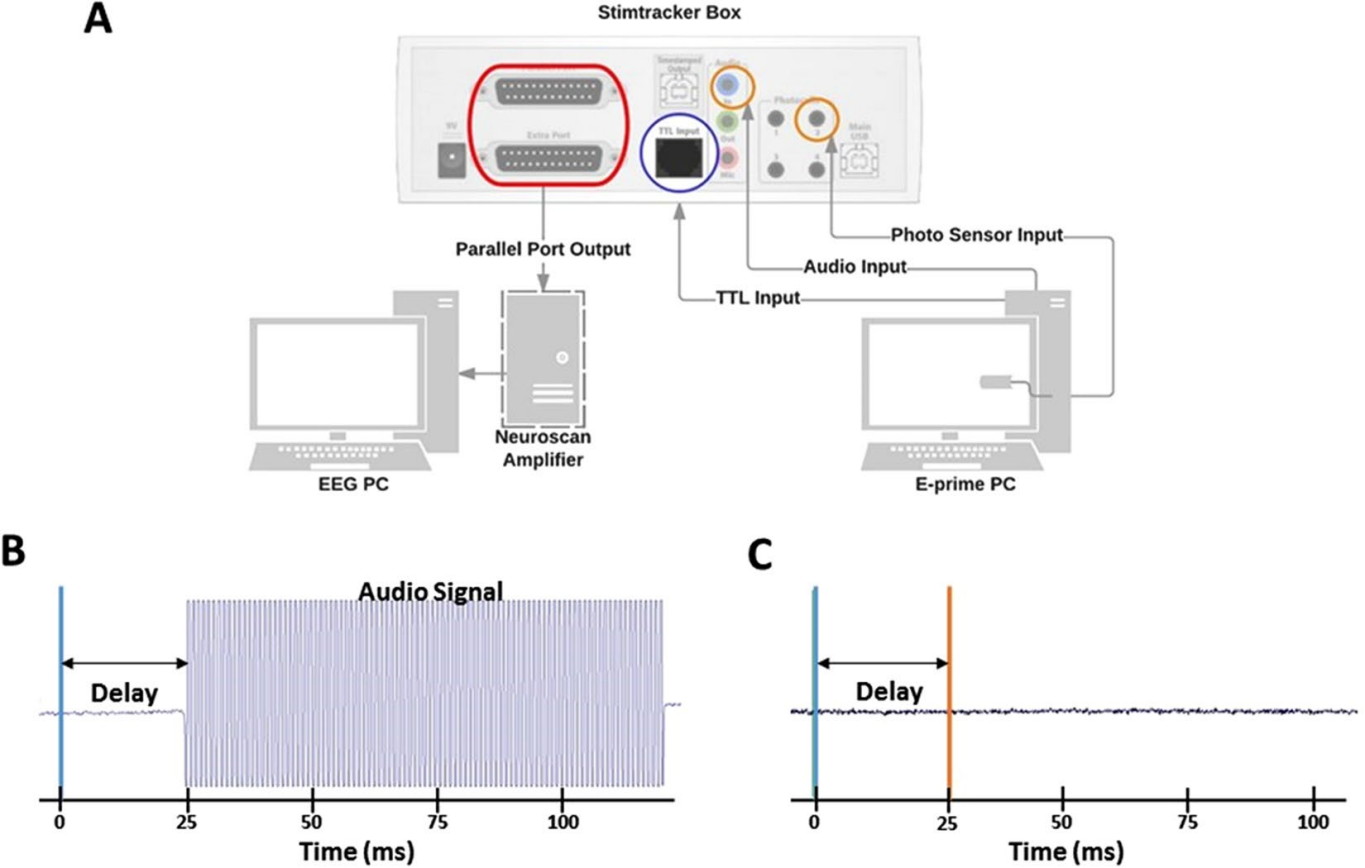
Measuring Time Delays in Task-based Multi-Site EEG Studies. (**A**) Setup of StimTracker system to measure the delay between the exact time of stimulus presentation (on E-Prime PC) and the time at which the stimulus onset was marked by the physiological data system (in EEG PC) (StimTracker diagram adapted from Cedrus website: http://cedrus.com/support/stimtracker/tn1460_st100_pins.htm) (**B**) Example of delay measurement when an audio signal is directly measured by the EEG system. (**C**) Example of auditory stimulus delay measurement with StimTracker system. The figure was drawn by authors using Lucidchart (Lucid Software Inc, UT, USA).

To assess the precision of StimTracker, a supplementary experiment was conducted. Two scenarios were contrasted using auditory stimuli. In the first scenario (Fig. 2B), the true onset time of an auditory stimulus was detected by directly connecting the audio signal to the EEG amplifier through bipolar EEG channels. This audio signal was attenuated to avoid amplifier saturation. In the second scenario (as shown in Fig. 2C), StimTracker was used to detect the true onset of the auditory stimulus by directly monitoring the audio output from the task computer. In both scenarios, the EEG computer records the event marker associated with the onset of the stimuli sent by the E-Prime computer (seen as a blue vertical line in Fig. 2B,C). Results showed that StimTracker could accurately measure the time delay in stimulus presentation.

In all subsequent tests, StimTracker was used to assess the described time delay across computer models/operating systems and monitors. During these tests, StimTracker was configured to receive timing information of stimuli (true stimuli onset and onset of triggers sent from E-Prime computer) and relay this information to the EEG system (Fig. 2A). To measure the true onset of a visual stimulus, a photosensor was used to detect a white visual stimulus on the monitor connected to the E-Prime computer. To measure the true onset of an auditory stimulus, an audio cable was connected from the task computer to StimTracker to detect auditory stimuli. To record the event marker sent by E-prime software to the EEG recording computer, the event marker was converted to a TTL signal using a DB25-to-RJ45 adapter. The time delay was then calculated as the “true onset of stimulus” minus the “actual onset marked in the EEG system”. A summary of the measured delay times are shown in Table 1 for a number of scenarios.

As illustrated in Table 1, the delay time associated with an auditory stimulus is much larger and more variable on a Windows 7 system (+/−2.93 ms) compared to a Windows XP system (+/−0.59 ms). Delays in the visual stimuli were not significantly different between the two operating systems. However, the delays were significantly different depending on the type of monitor and the location of the visual stimulus. Finally, the time delay of stimulus presentation was found to increase over sequential trials (up to 2 ms accumulation) before it resets.

Collectively, these findings suggest that multi-site research studies should be aware of these sources of variance in the data. Time delays in stimulus presentation should be accounted for to minimize inter-site variance. This can be done either by measuring and documenting the time delays associated with each equipment setup at each site using similar tests as described here, or by using the same equipment model, operating systems, and monitors across all sites.

### EEG acquisition parameters

Acquisition parameters should also be standardized across sites at the time of EEG collection and/or post-hoc, during data pre-processing.

In standardizing EEG parameters across sites, the site(s) with the most limited equipment condition (e.g., least number of EEG electrodes, lowest sampling rate, lack of flexibility in selection of reference electrode, etc) would dictate the lower boundary of EEG data acquisition parameters. Then, other sites could choose one of two strategies: (1) either set their acquisition parameters to this lower boundary, or (2) choose parameters that can later on (offline) be reduced to match this lower boundary. The latter approach has the advantage of maximizing the chance for future data integration with other studies that are not *a priori* planned. In general, the less restricted the acquisition parameters, the higher the probability of data reusability. However, caution should be exercised to not introduce other sources of variations in the experiment setup as a result of using different acquisition parameters. For example, let’s assume a scenario in which one study site has access to a 128-channel EEG system, while another has access to a 19-electrode system. In this case, the time allocated for EEG cap preparation may significantly differ between the two sites. As a result, the participants undergoing the 128-channel EEG recording may potentially experience more fatigue due to the long and uncomfortable EEG cap preparation procedure. This may then influence the subject’s performance if a cognitive task is involved. Therefore, to remedy this situation, EEG sites with access to 128 channel system may choose to prepare a subset of channels that are common with other sites. Alternatively, a quick questionnaire may be administered at all sites after each EEG session to rate subject’s fatigue at each site and use this information as covariate.

### Monitoring the data collection procedure

In addition to standardizing EEG data acquisition parameters, standardized strategies should be employed to monitor data acquisition across sites. These standardized strategies include, but are not limited to: ensuring a common check-list is followed at each site to minimize human error *during* EEG data collection (e.g., consistent EEG cap preparation procedure, lowering the electrode impedance to an *a priori* decided threshold level (e.g., <5 kOhm) and maintaining it over the entire recording session, selection of appropriate acquisition parameters, providing identical instructions to participants across sites, ensuring event triggers are encoded in EEG, etc). In the case of human errors or unexpected hardware issues (i.e., disconnected electrodes), detailed comments must be noted and logged at the time of the EEG session. Finally, if feasible, dedicated research personnel could visit all EEG sites early on in the data collection phase of the projects to ensure all sites follow the agreed upon SOPs.

### Quality control prior to data sharing

In multi-site studies that employ a common informatics platform for data sharing and archiving (as in CAN-BIND), the quality of data should be investigated prior to data sharing. This step ensures that each uploaded file adheres to the elected standards of data acquisition: for example, quality control step can ensure the planned acquisition parameters and data annotation are followed and data files are not corrupted. Additional quality control or data security steps may be considered as well. For example, EEG headers may be scanned for any patient information to ensure that uploaded data are de-indentified. A semi-automated data quality control script may be run to check each file for quality of recording (e.g. level of noise, disconnected electrodes, etc.) and notify sites if agreed upon standards are not followed.

### EEG data pre-processing

In CAN-BIND, we have thus far implemented two levels of standardized data pre-processing modules for study investigators (see below). Although investigators are not obliged to use these pipelines for all manuscripts, these methods serve as our core standards for data preprocessing. Any deviation from these standards must be scientifically justified.

#### Pre-Processing Module 1

The aim of the first data pre-processing module is to minimize raw data heterogeneity across sites and prepare the data for integration. If unique acquisitions systems are used between CAN-BIND sites it is crucial that the raw data files be re-configured into the same file format and composition. This can be done in MATLAB (The Mathworks, Inc., Natick, MA, USA) via the open-source EEGLAB toolbox^9^. During this process, the data is also downsampled (e.g., sampling rate and electrode montages are reduced) and re-referenced such that data from all sites are converted to have equivalent sampling rate, bandwidth, electrodes, reference, and event matrix. In CAN-BIND, this pre-processing step has thus far been conducted by dedicated research personnel and the converted data files are in EEGLAB format (*.set) which are shared on the Brain-CODE EEG platform.

An important step in this pre-processing stage is to address the variety of recording channels and their associated layouts. There are currently no established guidelines in place for integrating data between acquisition systems. When standard systems are used across acquisition systems (e.g., 10-10 EEG system), one way to address this issue is to find the closest equivalent electrodes between layouts (approach currently adapted in CAN-BIND projects). This approach has a downside when a standard montage (e.g., 10-10 EEG system) is not used across all or most sites. In such a case, a large proportion of electrodes may not have equivalents thereby resulting in a loss of spatial resolution and potentially impacting the types of EEG analyses that can be performed. For added accuracy, investigators may also choose to digitize the three-dimensional representation of the electrode layout and skull shape for every EEG recording session through commercially available digitizers (e.g. Polhemus Patriot digitizer). Including this information with each EEG recording could also provide a means to account for issues related to human error in proper EEG cap placement and improve accuracy in the interpretation of EEG outcomes.

#### Pre-Processing Module 2

**Figure 3:**
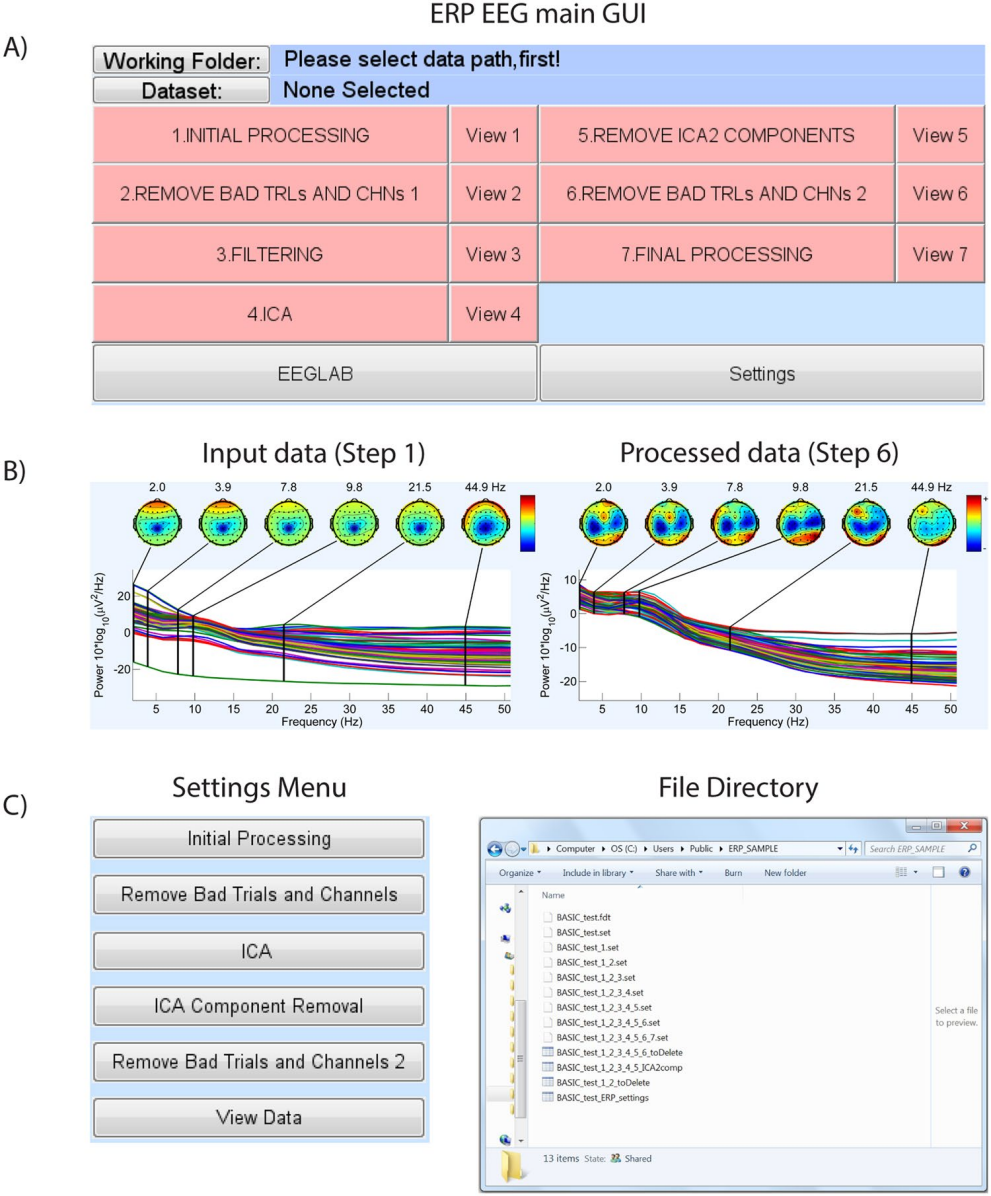
A Streamlined Toolbox for Multi-site EEG Data Processing and Archiving. (**A**) The main graphical user interface (GUI) of the ERPEEG toolbox, with 7 pre-processing steps. Through this main interface, users select the data (by clicking on Working Folder, and Dataset), and navigate through each preprocessing step. Clicking on a processing step opens a new GUI associated with that step, or runs that processing step. Steps that are completed turn green, and uncompleted steps remain red. (**B**) The view button (corresponding to each step) provides a visual summary of data cleaning processing (e.g., plots the power spectrum). This enables monitoring of data cleaning progress or detecting any major errors and data distortions. (**C**) The setting tab allows for selection of user-defined parameters for each step. (**D**) All intermediate steps (files created in completion of each step) are saved in the working folder following a standardized naming convention.

The aim of the second pre-processing module is to standardize and track EEG data through the noise removal procedure. For this purpose, to streamline the process of EEG data cleaning, we developed an open-source MATLAB application, ERPEEG toolbox, depicted in Fig. 3. This toolbox is developed in MATLAB (R2013a) and built using the EEGLAB platform (v.12.0.2.6b; ref. 9). A copy of this toolbox can be downloaded from www.tmseeg.com/multisiteprojects. The ERPEEG toolbox is created following the same framework as TMSEEG toolbox^10^. TMSEEG toolbox enables processing of EEG collected during Transcranial Magnetic Stimulation (TMS-EEG)^11^ (e.g., standardized processing of TMS evoked potentials or TEPs), while ERPEEG is intended for processing of resting-state EEG and ERPs (Event-related potentials).

This toolbox has a main interactive graphical user interface (GUI) (Fig. 3A) that allows users access to the dataset working folder, a sequential list of pre-processing procedures, and the settings menu. Clicking on each step opens another interactive GUI with several data visualization suites designed for each specific processing step. The order of data processing steps in ERPEEG is standardized, and is optimized towards improving performance of each processing step. For example, random and large amplitude artifacts are processed early in the pipeline in order to increase the performance of the independent component analysis (ICA) step later in the pipeline.

Following the initial step to load data and segment it into trials (Step 1), the workflow provides a GUI for *removing* data segments that are contaminated with random noise and cannot be easily de-noised. This step is particularly targeted towards the removal of channels and trials contaminated with large-amplitude or random noise sources that cannot be extracted easily through blind source estimation technique or filtering. The GUI permits interactive deletions of trials, channels and specific trials in a channel (Step 2) and keeps a log of deleted segments. Filtering can then be applied to exclude low and high frequency noise (Step 3). After these initial cleaning steps, blind source separation techniques such as ICA can then be used (Step 4, 5) to *extract* eye movements, eye blinks, electromyography artifacts, electrode discontinuity, and cardiac signals in order to recover the desired brain signals. At the completion of this step, a further interactive GUI enables a final data review and removal of trials and channels still contaminated with random noise (Step 6). Finally, the GUI provides users the options to interpolate the deleted channels and re-reference the data (Step 7).

**Figure 4:**
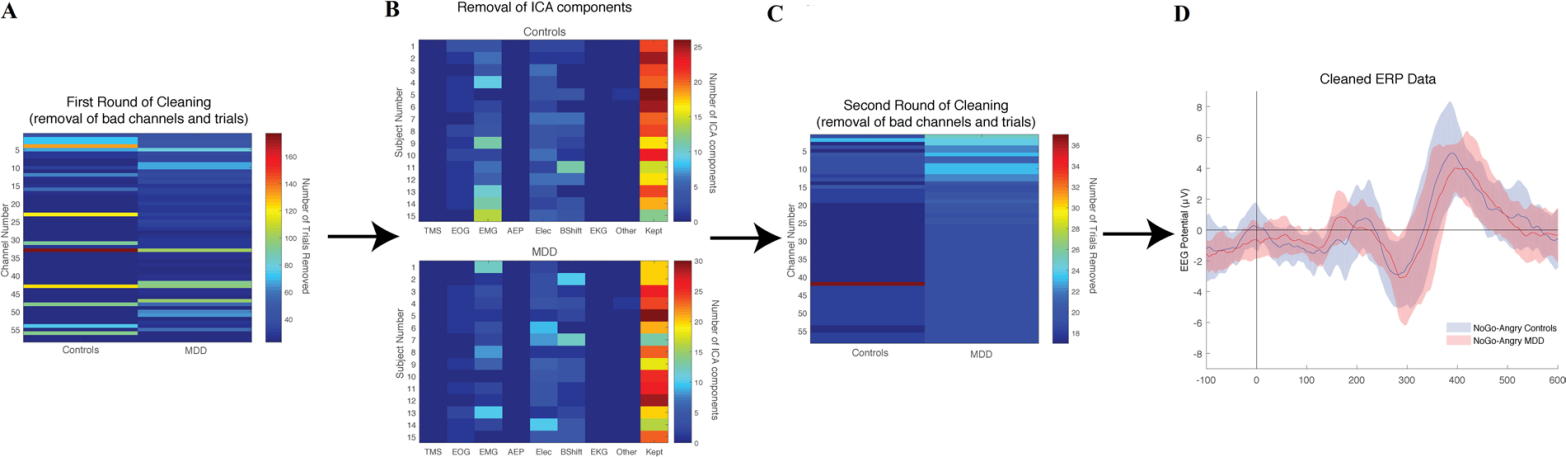
Illustration of Utility of the ERPEEG Toolbox with Pilot Data. The panels illustrate application of ERPEEG toolbox on pilot data from CAN-BIND project 1. Pilot data include EEG collected during affective Go/noGo task in 15 healthy controls and 15 MDD patients. Raw data were imported into MATLAB via EEGLAB^9^, epoched around onset of each stimulus (e.g., Go or NoGo cue superimposed on angry, happy or neutral faces), and preprocessed with ERPEEG toolbox. Panels A to C illustrate the outcomes of steps 2, 5, and 6 of ERPEEG toolbox, respectively, compared visually between MDD and control groups. Panel D, illustrates the final cleaned ERP, outcome of step 7, compared between MDD and controls for NoGo angry face condition. (**A**,**C**) In both panels, x-axes depict two groups of controls and MDD, and y-axes depict channel number, while colors illustrate number of trials removed for each channel averaged across subjects following step 2 (panel A), and step 6 (panel C). (**B**) In both panels, the x-axis depicts the component type (e.g., component related to eye blinks, bad electrodes, EKG, etc), y-axis shows the subject number, and the colors depict number of components removed in control (top) and MDD (bottom). The matrix of deleted trials depicted in each of these images can be used to systematically compare the data processing across controls and patients, but also across different investigators, and projects.

The ERPEEG toolbox contains several important features for standardized EEG data pre-processing. First, it incorporates interactive data visualization capabilities (Fig. 3B), allowing the user to visualize the data at each step of the workflow and verifying the effectiveness of the data cleaning procedure. Second, intermediate datasets are saved after each processing step along with other important meta-information such as the deleted trials and channels, and the removed artifacts (Fig. 3C, File Directory). This enables the user to easily revert to a previous step in the workflow, check the output of each step, and create a database of selected artifacts. Third, ERPEEG is a flexible platform. It allows for basic customization through the settings menu (Fig. 3C) while providing a modular structure for advanced users to modify the order of processing or incorporate additional steps to accommodate processing of different EEG projects. Finally, parameters selected through the setting menu are saved in a separate MATLAB file. This enables future replication or assessment of the data pre-processing steps. The utility of ERPEEG toolbox is illustrated in preliminary data (Fig. 4) from CAN-BIND 1 project involving EEG recorded during Go/NoGo task in fifteen healthy controls and fifteen MDD subjects. An early version of ERPEEG toolbox was also successfully used in a non CAN-BIND project^12^.

### EEG Features

EEG features refer to EEG neural markers (e.g., power of alpha oscillations) that may be used to differentiate between two or more study groups (e.g., patients with depression from healthy controls, responders to treatment from non-responders). Numerous linear and non-linear algorithms are designed to extract neural markers from pre-processed EEG. However, these algorithms may involve subjective selection of several parameters by the experimenter (e.g., selection of time windows, frequency bands, or methods of transferring signals from one domain to another, or sensor to source space). To prevent subjectivity, the data processing algorithms should be described in great detail within the corresponding manuscript to enable future replication or to diagnose the cause for a lack of replication in future studies. Specifically, any subjective parameters should be reported. Alternatively, EEG feature algorithms and associated parameters may be shared on an accessible informatics platform.

### Choice of statistical frameworks and machine learning algorithms

Another source of variance between EEG studies is the choice of statistical frameworks. In particular, the methodologies used for correcting multiple comparisons or evaluating the accuracy of machine learning algorithms are among factors that can impact study outcomes. In multi-site multi-investigator studies, these factors can lead to different findings between different groups of investigators.

#### Addressing Multiple Comparisons

Electrophysiological brain response can be characterized across several dimensions (e.g., frequency, time, and space). Typical analysis of multidimensional data evaluates the significance of “active” elements (voxels), i.e., voxels that meet a certain threshold of statistical significance (generally t-score or z-score) compared to other voxels in the dataset. Traditional analysis on a voxel-wise basis involves a very large number of comparisons and runs the risk of Type I errors (false positives). This is known as the multiple comparisons problem (MCP). Several analytical frameworks can be chosen to address the MCP in neuroimaging studies. Classically, MCP can be addressed using voxel-wise methods such as the Bonferroni correction and others^13^. However, these methods are thought to be unnecessarily conservative and may reduce the sensitivity of detecting a true effect. This can alternatively lead to Type II errors (false negatives).

To address such Type II errors of these conservative approaches, cluster-based thresholding frameworks have been introduced^14–16^. Cluster-based frameworks take advantage of smoothness across one or more dimensions in EEG and related neuroimaging data by grouping neighboring active (or statistically significant) voxels to represent *a significant cluster*. In EEG recordings, spatial smoothness (i.e., across sensors) results from the inherent presence of volume conduction. This is particularly prominent in high-density EEG recording (e.g., >32 electrodes). Smoothness may also be present in the time or frequency domain when the sampling rate of data acquisition is higher than the dynamics of scalp EEG recordings. Therefore, neurophysiologically meaningful differences often extend to adjacent voxels, across one or more dimensions, forming clusters of significant values.

**Figure 5:**
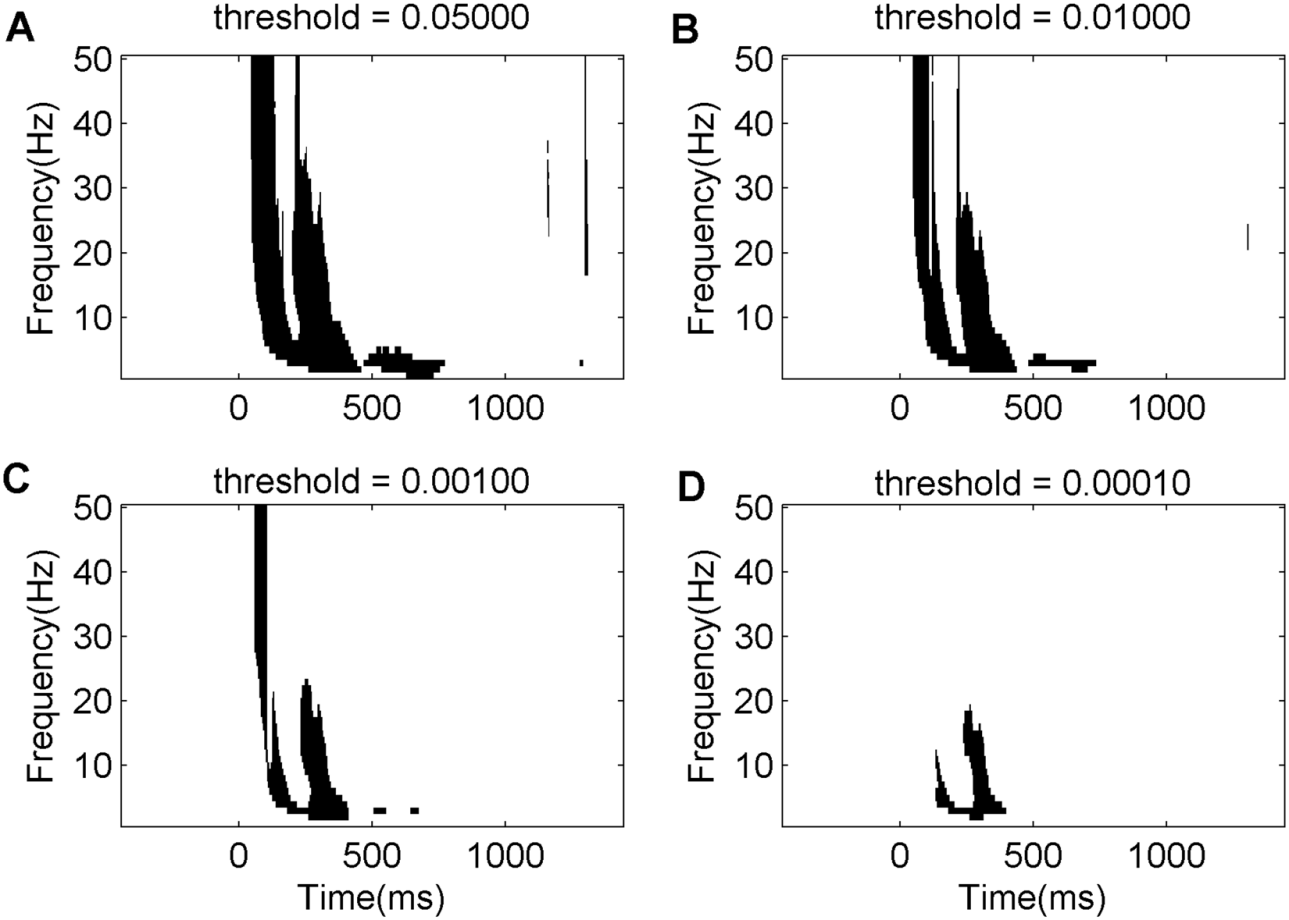
Illustration of Utility of Unbiased Cluster Estimation Technique. Panels A to D illustrate the impact of varying threshold statistic on the outcome of cluster estimation. The figure is adapted with permission from Frehlich *et al*.^17^. Data were collected from EEG channel CZ and compared statistically between two conditions. Y-axses represent frequency in Hertz and x-axes time in milliseconds with time 0 indicating application of a transcranial magnetic stimulation pulse. Each dark pixel denotes a voxel in the time-frequency space that meets a statistical criteria given by the threshold statistic. The panels illustrate how changing the threshold alters the number, size of clusters, and interpretation of the findings in time-frequency space.

Cluster-based frameworks are typically implemented in two steps: 1) setting a threshold-level (e.g., p < 0.05 or p < 0.01) for detecting statistically-significant cluster of active, neighboring voxels, and 2) calculating the p-value for each cluster by evaluating the probability of the cluster occurring by chance. To assess this probability, a *cluster measure* is computed for each detected cluster. Common cluster measures include: *cluster-size* that includes the number of active voxels in a cluster; *cluster-maximum* that is the maximum intensity voxel (e.g., highest t statistics) within a cluster, and recently a hybrid approach, *cluster-mass*, that involves summing all intensity values (e.g., summing all t statistics) within a cluster. However, these clustering methods have a well-known caveat: setting the initial threshold (e.g., p < 0.05 or p < 0.01) can have a profound effect on the resulting clusters and their significance, thereby introducing user bias. For example, when comparing the time-frequency maps of evoked potentials between two conditions, as depicted in Fig. 5, varying the significance threshold value from p value of 0.05 to 0.0001 can significantly impact the outcome measures. This can lead to discrepancy in interpretation of findings between researchers. To account for this, we have recently introduced the Unbiased Cluster Estimation (UCE)^17^ method, a threshold-free extension to traditional cluster-based analysis to avoid user bias. Our approach calculates cluster values at all relevant p-values and combines them using an averaged approach, removing the reliance on thresholding and considering information from multiple threshold levels. An early version of UCE was successfully used in a non CAN-BIND project^18^. In multi-site, multi-investigator, and multi-project initiatives such as CAN-BIND that involves several parallel projects which share common overarching aims (e.g., identification of biomarkers of response to antidepressants), utility of such parameter free statistical frameworks enable meaningful comparisons of outcomes across projects.

#### Prediction Accuracy in Machine Learning Algorithms

In recent years, there has been a growing interest in using machine learning algorithms to predict health outcomes or classify neurological disorders. Among sources of variation in different EEG studies are the features used for classification and the method used to assess the prediction accuracy from machine learning^19^. Supervised machine learning is typically implemented using the following steps: 1) Features are computed from pre-processed EEG data. 2) Features are then used with a classifier and the classifier is trained to map feature vectors to an appropriate label (e.g., healthy or depression). The classifier is then used to predict the label of new subjects based on their features. 3) Classification accuracy of this algorithm is assessed. A standard method for assessing the accuracy of the trained classifier is leave-n-out (e.g., n = 1) cross-validation. Data are divided into small equally sized subsets, and the classifier is trained on all subsets except n (e.g., one), the randomly chosen validation subset, that is used to assess the accuracy of the classifier. The training and the test data sets are permuted, and the process is repeated until all combinations are exhausted. The average accuracy across iterations is then reported as the accuracy of the classifier. Variations in the use of cross-validation (e.g., record-wise vs. subject-wise cross-validation) can significantly impact the prediction accuracy of machine learning algorithms^19^. For example, as described in details by Saeb *et al*.^19^, in record-wise cross-validation, data are randomly divided into training and test folds irrespective of subject label. This approach will lead to presence of data from the same subject in both the test and the training set. It can be shown that record-wise cross-validation leads to significant overestimation of the accuracy compared to subject-wise comparisons. Therefore, the testing methodology used for machine classification should be carefully detailed and reported. The availability of large volumes of data through multi-site studies also provides the opportunity to further validate the accuracy of the trained algorithms in an independent cohort. This independent cohort could be a locked dataset (e.g., data from one site) that is independent from the dataset used to train and cross-validate the model and that can be set aside for this purpose.

### Data Archiving

To enable replication of results or to understand future challenges in replicating original findings, archiving the outputs of intermediate analysis steps is recommended. As described in section 6, our customized pre-processing toolbox saves and logs the output of each EEG pre-processing step, along with the meta-data summarizing details of each pre-processing step (e.g., information on deleted segments, filter parameters, rejected artifact components, etc). Any or all of these intermediate files may be saved on the informatics platform for future reference and assessment of impact of data pre-processing choices on the EEG outcomes. In cases where multiple modalities are involved (e.g., EEG and fMRI), similar naming convention should be used for equivalent datasets to enable integration and cross-comparisons.

### Knowledge Translation

In large-scale multi-site, multi-platform, and multi-investigator initiatives such as CAN-BIND, early and frequent conversations among investigators are needed to streamline the process of research knowledge translation. We propose that knowledge translation can be considered in two stages: Stage 1 involves dissemination of scientific findings, including developed methodologies (such as present article) and research articles that originate from data collected through the projects. Stage 2 involves translation of findings into everyday clinical practice, including again both the developed methodology to translate findings and the actual practical translation of the research findings. In Stage 1, the priority of the various research hypotheses, the sequence in which research questions must be addressed when using common EEG datasets and the dissemination of results via peer-reviewed journal articles must be reached by consensus. Specifically, in multi-platform projects that ultimately involve integration of data across multiple platforms (e.g., integrating EEG, fMRI, clinical, and genetic data, or integrating data collected in humans and animals), regular communication should take place between leaders of platforms on issues surrounding the standardization process to ensure that data and outcomes can be ultimately integrated. For example, in CAN-BIND, a web-based discussion platform (e.g., Basecamp) is utilized through which summary of monthly calls and meetings are shared and archived in a centralized fashion and made available to all participating sites and investigators. Similarly, methods and directions to be followed for Stage 2 of knowledge translation (e.g., handling of intellectual properties, commercialization plans) should be discussed early on and planned out by effective communication among investigators.

## Discussion

Standardization of data collection, analysis, and handling is at the core of multi-site, multi-investigator, and multi-project initiatives such as CAN-BIND that involve capturing and integrating data across multiple data platforms (e.g. clinical, EEG, neuroimaging, and molecular). Big data collected by a research network will ultimately be accessed by numerous research scientists and data analysts. Reproducibility and the ability to preserve and integrate study outcomes across projects and investigators are therefore integral components of the network and major determinants of its overall impact on improving human condition. In this paper, we described the measures taken and knowledge gained during standardization of the EEG platform of CAN-BIND. Many of the concepts and data standardization approaches introduced in this manuscript can be extended to other data platforms and other EEG multi-site studies.

In this paper, we described potential sources of variance across multiple stages of a research study when EEG data is collected across several sites. We described factors in study design that need careful consideration to minimize bias in research results across sites. Moreover, examples were provided of the types of data variability introduced due to differences in recording equipment. In the context of the CAN-BIND multi-site project 1^4^, differences in equipment was a challenge in the collection of task-based EEG (i.e., ERPs) data. We introduced a streamlined EEG preprocessing toolbox to standardize, assess, and track the impact of EEG data pre-processing decisions and subjectivity in EEG data cleaning on study outcomes. Finally, we drew attention to the impact of statistical frameworks on study outcome measures and introduced specific strategies aimed to attenuate such impacts.

The growing trend in multi-site research studies featuring integration of unstructured data across multiple institutions is likely here to stay. These trends are in part motivated by challenges in replicating research outcomes across laboratories, and the lack of generalizability of research outcomes derived from underpowered studies. While multi-site research projects aim to address these challenges, the benefits of such initiatives may be hindered by several sources of inter-site variance discussed in this article. This may be an issue in the near future because subsequent to the establishment of independent informatics platforms and collection of data through multi-site trials, the scientific community will soon begin to compare findings between the platforms and trials. The integrity and shelf life of biological data depends on the quality, homogeneity, and proper documentation and handling of data and data parameters across investigators and laboratories. Therefore, timely discussion and establishment of recognized consensus and standards in EEG studies for wide-scale adoption would increase the likelihood of successful data integration and replication across informatics platforms. We propose that a comprehensive annotation of data and archiving of meta-data across all stages of a research study will allow for offline data standardization and enable re-usability of the data in novel analyses beyond the goals of the original study.
